# Towards interactive AI-authoring with prototypical few-shot classifiers in histopathology

**DOI:** 10.1016/j.jpi.2024.100388

**Published:** 2024-06-06

**Authors:** Petr Kuritcyn, Rosalie Kletzander, Sophia Eisenberg, Thomas Wittenberg, Volker Bruns, Katja Evert, Felix Keil, Paul K. Ziegler, Katrin Bankov, Peter Wild, Markus Eckstein, Arndt Hartmann, Carol I. Geppert, Michaela Benz

**Affiliations:** a Fraunhofer IIS, Fraunhofer Institute for Integrated Circuits IIS, Medical Image Analysis Group, Erlangen, Germany; b Institute of Pathology, University of Regensburg, Regensburg, Germany; c Frankfurt Cancer Institute (FCI), Goethe University Frankfurt, Frankfurt am Main, Germany; d Dr. Senckenberg Institute of Pathology, University Hospital Frankfurt, Goethe University Frankfurt, Frankfurt am Main, Germany; e Frankfurt Institute for Advanced Studies (FIAS), Frankfurt am Main, Germany; f Institute of Pathology, University Hospital Erlangen, FAU Erlangen-Nuremberg, Erlangen, Germany; g Comprehensive Cancer Center Erlangen-EMN (CCC), University Hospital Erlangen, FAU Erlangen-Nuremberg, Erlangen, Germany; h Charité – Universitätsmedizin Berlin, corporate member of Freie Universität Berlin and Humboldt-Universität zu Berlin, Department of Pediatric Oncology and Hematology, Germany

**Keywords:** Digital pathology, Few-shot learning, Prototypical networks, Data augmentation, Tissue classification, Colon adenocarcinoma, Urothelial carcinoma

## Abstract

A vast multitude of tasks in histopathology could potentially benefit from the support of artificial intelligence (AI). Many examples have been shown in the literature and first commercial products with FDA or CE-IVDR clearance are available. However, two key challenges remain: (1) a scarcity of thoroughly annotated images, respectively the laboriousness of this task, and (2) the creation of robust models that can cope with the data heterogeneity in the field (domain generalization). In this work, we investigate how the combination of prototypical few-shot classification models and data augmentation can address both of these challenges. Based on annotated data sets that include multiple centers, multiple scanners, and two tumor entities, we examine the robustness and the adaptability of few-shot classifiers in multiple scenarios. We demonstrate that data from one scanner and one site are sufficient to train robust few-shot classification models by applying domain-specific data augmentation. The models achieved classification performance of around 90% on a multiscanner and multicenter database, which is on par with the accuracy achieved on the primary single-center single-scanner data. Various convolutional neural network (CNN) architectures can be used for feature extraction in the few-shot model. A comparison of nine state-of-the-art architectures yielded that EfficientNet B0 provides the best trade-off between accuracy and inference time. The classification of prototypical few-shot models directly relies on class prototypes derived from example images of each class. Therefore, we investigated the influence of prototypes originating from images from different scanners and evaluated their performance also on the multiscanner database. Again, our few-shot model showed a stable performance with an average absolute deviation in accuracy compared to the primary prototypes of 1.8% points. Finally, we examined the adaptability to a new tumor entity: classification of tissue sections containing urothelial carcinoma into normal, tumor, and necrotic regions. Only three annotations per subclass (e.g., muscle and adipose tissue are subclasses of normal tissue) were provided to adapt the few-shot model, which obtained an overall accuracy of 93.6%. These results demonstrate that prototypical few-shot classification is an ideal technology for realizing an interactive AI authoring system as it only requires few annotations and can be adapted to new tasks without involving retraining of the underlying feature extraction CNN, which would in turn require a selection of hyper-parameters based on data science expert knowledge. Similarly, it can be regarded as a guided annotation system. To this end, we realized a workflow and user interface that targets non-technical users.

## Introduction

**Fig. 1:**
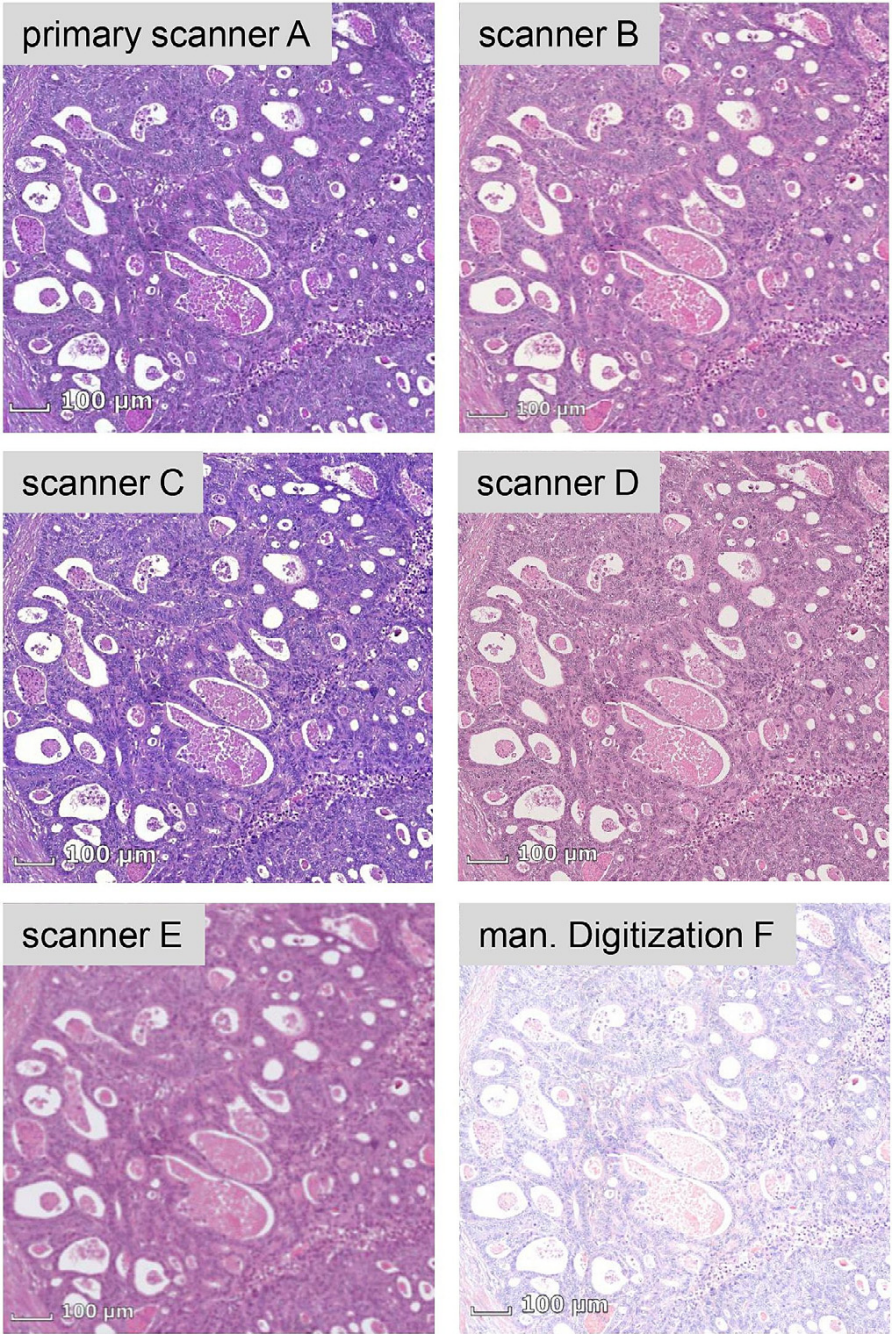
Image cut-outs of WSIs that were created with five different automated microscopic scanners (primary scanner A, scanner B–E) and by a manual digitization (F) procedure using a real-time stitching software from the same hematoxylin and eosin-stained tissue section showing an example of colon cancer. The introduced domain shift is clearly visible.

Over the last decade, digitization has increasingly spread into pathology. In the scientific field, it is now common practice to create digital so-called whole-slide images (WSIs) from tissue sections prepared on glass slides instead of examining them directly under a microscope. The availability of digital scans, in turn, allows for the introduction of computer-aided assistance systems. Numerous deep neural network (DNN) approaches have been developed and investigated for various applications^2^^,^^3^^,^^22^^,^^32^ such as metastasis detection, tissue classification, or nuclei segmentation. However, there is a huge variety of organs, disease types, biomarkers, and stains, and therefore still numerous applications are not yet covered. As the creation of spatially annotated data sets is very time-consuming, one challenge that typically arises in the development of new DNNs is the scarcity of labeled data. Another challenge is the domain shift introduced by differences in the digitization and staining process of tissue sections. For instance, the same tissue section digitized by different scanners lead to WSIs with significant variance in color appearance (Fig. 1). Such variations may also be caused by differences in the staining process between different clinics. DNNs need to be able to cope with this variance when deployed into the field. Therefore, our goal was to develop an easily adaptable and robust classifier for tissue analysis in histopathology and integrate it into a workflow with a user interface that supports even non-technical users in creating their own adapted artificial intelligence (AI) classifier.

### Related work

One common approach to overcoming the challenge of scarcity of labeled training data is transfer learning (TL). TL aims to transfer knowledge from already solved tasks to a new task^17^ and so reduces the required amount of training data for the new task. Kim et al. conducted a survey on TL with pre-trained convolutional neural network (CNN) models for medical image analysis.^17^ They categorized TL approaches into two main categories, which they denoted as “fine-tuned” and “feature extractor”. Their category “fine-tuned” contains all approaches that involve a retraining of all or a subset of the parameters of the pre-trained model. Approaches of this category always fine-tune at least a subset of the parameters of the convolutional layers. In contrast, “feature extractor” approaches freeze all convolutional layers. In other words, the feature extractor part of the pre-trained CNN model is applied without changes to the new task and only the classification is adapted. Kim et al. distinguish between two sub-categories: in the first one, the pre-trained model's fully connected layers, which are responsible for the classification, are fine-tuned; in the second one, the classification in the new task is performed with a classical machine learning algorithm such as a Random Forest classifier.^17^ Kim et al. recommended the feature extractor approach, especially when the characteristics of the medical images of the new task are similar to those that the original model was trained on.

Few-shot learning (FSL) methods in general also follow the principle of TL, but they differ in the training mechanism^21^: typically, a meta-learning^14^ or episodic-training mechanism^26^ is applied. Within this work, the term FSL is used to summarize all concepts for training DNNs using only a few annotated examples and applying a meta-learning or episodic-training mechanism. In addition, only classification models are examined within this work, and these are referred to as few-shot models or few-shot classifiers if they have been trained using a FSL approach.

A comprehensive introduction to meta-learning is provided by Hospedales et al.^14^ Meta-learning can be understood as “learning to learn”. In contrast to standard learning procedures, where model predictions are optimized over multiple data instances, in meta-learning, the model is optimized over multiple tasks. According to Hospedales et al., two training phases can be distinguished: a base training on a set of source tasks and afterwards a meta-training step with additional tasks in order to learn a meta-algorithm that allows the model to learn how to adapt to new tasks.

The episodic-training mechanism also relies on tasks: given a few labeled support instances, unknown queries have to be predicted. This learning scheme is typically applied for metric-learning-based FSL methods that employ a learning-to-compare concept.^21^ They aim at learning representations (also called embedding) that generalize across different tasks. This is implemented by a direct comparison between query and support images during the episodic training. One popular approach are the Prototypical Networks introduced by Snell et al.^26^ Classes are represented by prototypes in the embedding space and query images are assigned the label of the nearest prototype using the squared euclidean distance. Besides using fixed metrics like in Snell et al.,^26^ another option is to directly train a deep non-linear distance metric, which is for instance realized in the Relation Network introduced by Sung et al.^27^ One key advantage of these metric-based FSL methods is that they can be adapted to a new task without retraining the underlying DNN. Instead, only the class prototypes have to be exchanged. Therefore, we have chosen this approach for our work.

An extensive comparison of various FSL methods on natural image data sets is provided by Li et al.^21^ Few-shot methods have also been successfully applied in histopathology. Rijthoven et al.^33^ combined prototypical few-shot networks with image retrieval and object detection in order to detect objects and tissue components within WSIs, in particular healthy glands in colon biopsies or ductal carcinoma in situ in breast tissue sections. They used an encoder network from Tellez et al. that was trained simultaneously on four tasks.^31^ Wang et al. applied FSL for tumor classification on microscopic images of breast cancer tissue.^34^ They investigated different feature map fusion strategies^34^ using a wide residual network (WRN)^36^ as a feature extractor and showed that it is beneficial to combine features from different convolutional blocks from the WRN. Chao and Belanger applied FSL, specifically an adapted version of the model-agnostic meta-learning (MAML) method,^9^ in order to classify histopathology images from tumor biopsy samples with and without whole-genome doubling.^4^ Their goal was to train a DNN that is able to generalize across different cancer types, including liver or colon cancer. They demonstrated that their MAML classifier obtained superior results compared to a baseline CNN classifier on a test set with different cancer types. Moreover, they compared the classifier performance on images that were disturbed by brightness and resolution changes. To ensure the robustness of their classifier, they introduced data augmentation—random vertical/horizontal flips and color jitter—during the training process.

Data augmentation is one common approach to countering domain shift introduced by differences in the digitization or staining process. Augmentations are applied to the images of the training set during the training process to simulate expected variance in the test images. Typical augmentations are the artificial variation of hue and saturation values or modulation of Gaussian noise onto the original images. Tellez et al. introduced a specific augmentation for hematoxylin and eosin (HE)-stained images, where the first step is a transformation into a stain-specific color space.^30^ For each augmentation, parameters such as the magnitude of the distortion have to be chosen. Moreover, several augmentations can be applied in conjunction with an image or only separately. Therefore, different strategies for selecting augmentation settings have been proposed in the literature, e.g., Randaugment,^6^ TrivialAugment,^24^ or a data-driven method specifically for HE-stained images.^23^ Alternatively, generative adversarial networks can be used for data augmentation.^1^ Another approach to coping with variances in the data is to normalize all images before classifying them. A comprehensive comparison of the effects of data augmentation and stain color normalization on different tasks, including metastasis detection, is provided by Tellez et al.^30^ However, one huge disadvantage of this approach, as well as style transfer GANs, is that they are applied at run time and thus increase the overall computation time per slide. Therefore, within this work, data augmentation was applied, which only increases the training time, but not the inference throughput.

### Contributions

The contribution of this work is 3-fold: (1) creation of robust prototypical few-shot models for tissue type classification in histopathology, (2) comprehensive evaluation on a multiscanner and multicenter database, and (3) adaptation of a prototypical few-shot model to a new task with a simple and user-friendly workflow.

(1) We trained several prototypical few-shot models with different network architectures, applying data augmentation for tissue type classification on colon tissue sections. During training, only data from one scanner and one clinic was used. (2) To investigate the robustness of the models against domain shifts, we evaluated them on multiscanner and multicenter data sets. Here, we compared different variations, e.g., training with and without data augmentation, or using prototypes derived from data from different scanners. (3) We created a workflow with a graphical user interface for adapting a prototypical few-shot model to a new task and performed an evaluation on an additional data set consisting of tissue sections with urothelial cancer.

## Material and methods

This section provides a detailed description of the multiple data sets used in this work and the applied methods.

### Data

Most data originate from HE-stained tissue sections of the primary tumors from patients with colorectal adenocarcinoma. The one exception is a separate data set, which originates from HE-stained tissue sections from patients with urothelial carcinoma. As the tissue sections were digitized with scanners by different vendors, the resolution varies between 0.17 and 0.35 μm/pixel. A set of tissue classes was annotated manually in the WSIs and approved by an experienced pathologist.

#### Colon adenocarcinoma primary data set

**Fig. 2:**
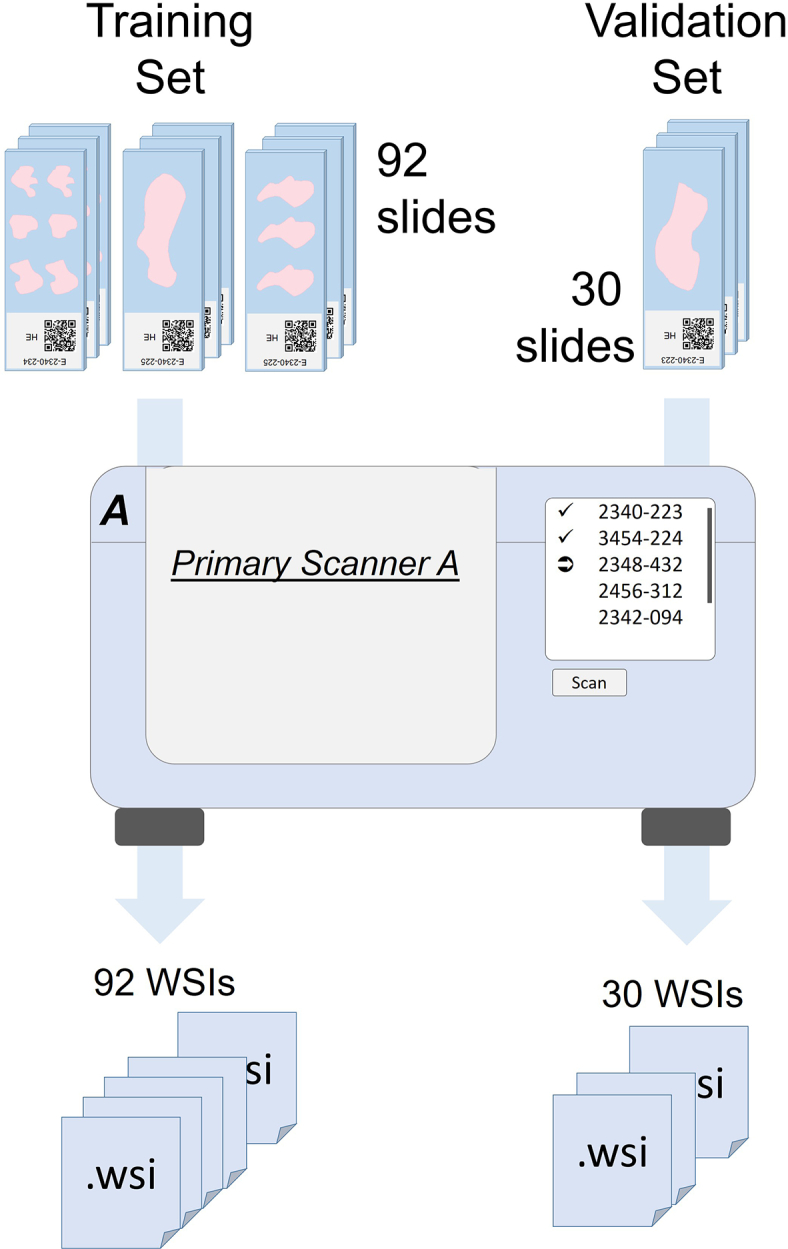
The training and validation sets for all models within this work originate from one center (University Hospital Erlangen, Center 1) and are digitized with the same scanner (*primary scanner*).

The primary data set consists of 161 HE-stained tissue sections from patients with colon adenocarcinoma of various grades from the University Hospital in Erlangen (Center 1), Germany. Slides were digitized using a *3DHISTECH Pannoramic 250* slide scanner (scanner A) with an objective magnification of 20 and a resolution of 0.22 × 0.22 μm/pixel. Annotations in these WSIs cover the seven tissue classes: *tumor cells*, *muscle tissue*, *connective tissue combined with adipose tissue*, *mucosa*, *necrosis*, *inflammation*, and *mucus*. Based on these annotations, labeled image patches of pixel size 224 × 224 were extracted. Image patches with an insufficient overlap with the annotations (less than 85%) or patches that contain mainly background were discarded. The primary data was split into four disjoint sets: training (92 WSIs, 2,173,515 patches), validation (30 WSIs, 719,010 patches), test (30 WSIs, 1,381,316 patches), and adaptation set (9 WSIs, 344,926 patches). Image patches originating from one WSI are only contained in one set. All models within this work were trained on this training set and model selection was performed based on this validation set (Fig. 2).

#### Colon adenocarcinoma multiscanner data set

All tissue sections from the primary test set (30 slides) and adaptation set (9 slides) were digitized with four additional automated microscopic scanners: Precipoint M8 (scanner B), Fraunhofer Scube (scanner C), Hamamatsu Nanozoomer S210 (scanner D), and Hamamatsu Nanozoomer S360 (scanner E). Moreover, they were digitized manually (F) using a real-time stitching software (Fraunhofer iSTIX) (Fig. 1). In order to achieve this, a camera is mounted on a standard microscope and connected to a computer. The microscope stage is moved manually, while the glass slide is traversed systematically. At the same time, the software stitches the images from the video stream in real time, resulting in one WSI representing the glass slide. The data set (F) was included as a special challenge and sub-optimal settings for imaging conditions like saturation were chosen during digitization (Fig. 1).

**Fig. 3:**
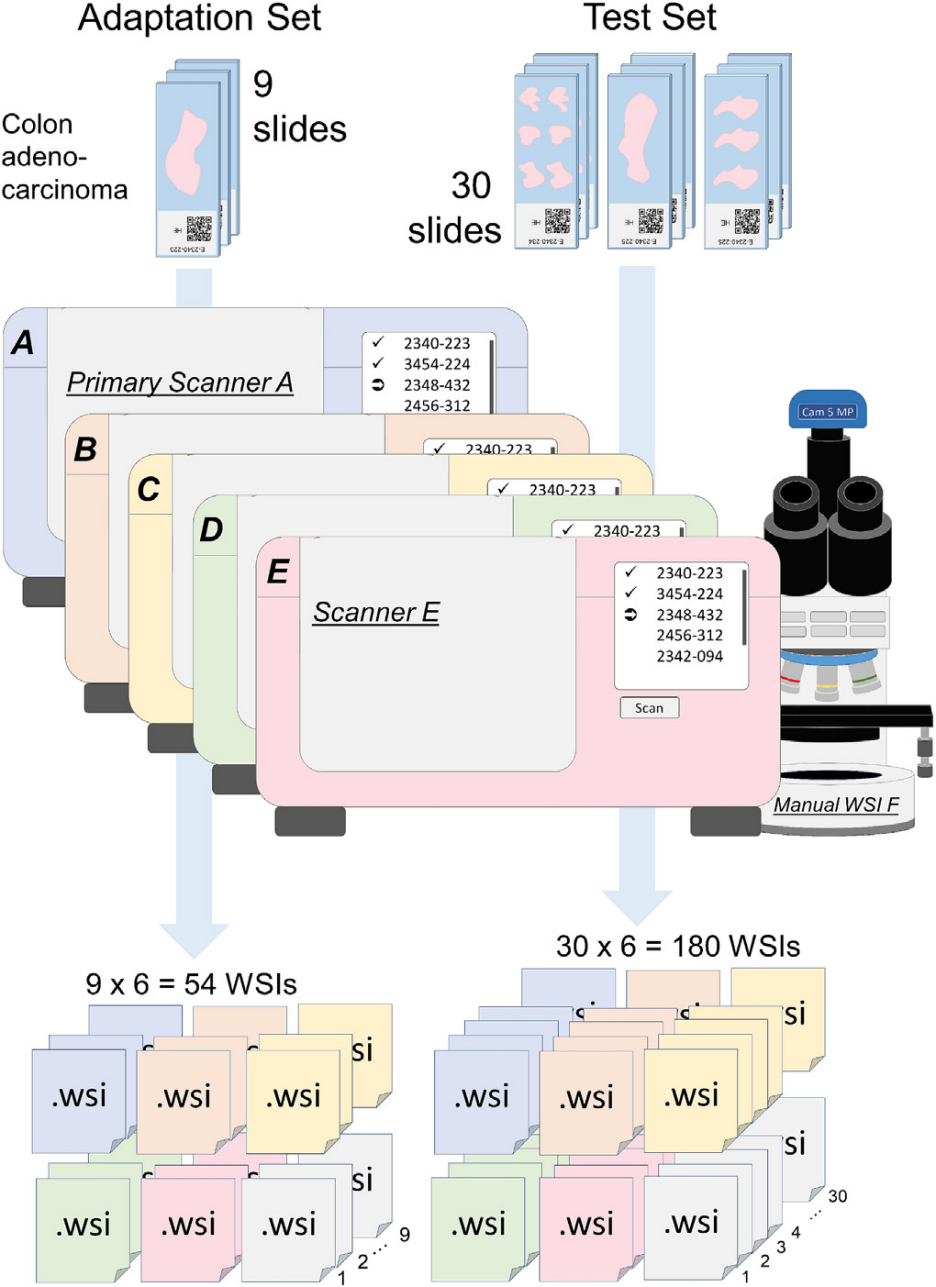
Overview of the multiscanner data set. It consists of six different test and adaptation sets.

Each WSI was aligned to the WSI obtained by the primary scanner so that the annotations of the tissue classes could be transferred to it as described in Kuritcyn et al.^20^ Fig. 3 provides an overview of the resulting multiscanner data set.

#### Colon adenocarcinoma multicenter data set

The primary data set as well as the multiscanner data set were generated from tissue sections that stem from only one site (University Hospital Erlangen, Center 1). The multicenter data set, however, was created with tissue sections from different sites: WSIs were provided by the Institute of Pathology in Regensburg (Center 2) and the Dr. Senckenberg Institute of Pathology in Frankfurt (Center 3). The tissue sections of Center 2 were digitized using a *3DHISTECH Pannoramic 1000 Flash RX* slide scanner with an objective magnification of 20 and a resolution of 0.24 × 0.24 μm/pixel. The tissue sections of Center 3 were digitized using a *3DHISTECH Pannoramic Scan II* slide scanner with an objective magnification of 20 and a resolution of 0.27 × 0.27 μm/pixel. Moreover, additional WSIs were downloaded from The Cancer Genome Atlas Program (TCGA). These WSIs originate from different sites but are jointly denoted here as (Multi) Center 4. The WSIs from these extra sites were also manually annotated, using the same tissue classes and criteria employed in the primary data set, and again approved by an experienced pathologist. Table 1 gives an overview of the resulting data sets.

**Table 1:** For each center, the number of labeled patches contained in the respective test set and the number of WSIs from which the patches were extracted is provided. The resolution of the WSIs differs between the centers and within (Multi) Center 4 because different scanners were used for the digitization.

	# WSIs	# patches	Resolution [μm/pixel]
Center 1 (Erlangen)	30	1,381,316	0.221
Center 2 (Regensburg)	16	191,582	0.243
Center 3 (Frankfurt)	46	364,295	0.275
(Multi)Center 4 (TCGA)	48	303,174	0.233–0.253

#### Urothelial cancer data set

This data set originates from 55 HE-stained tissue sections containing urothelial carcinomas from a patient cohort from the University Hospital in Erlangen. Slides were digitized using a *3DHISTECH Pannoramic 1000* slide scanner. Within the WSIs, tumor, healthy, and necrotic regions were annotated manually and approved by an experienced pathologist. Six different subtypes of urothelial carcinomas were included: neuroendrocrine, sarcomatoid, plasmacytoid, micropapillary, squamous, and NOS (“not otherwise specified”). The class *healthy* consists of the subclasses *connective tissue*, *adipose tissue*, *muscle tissue,* and *inflammation*. The set was split into an adaptation set (18 WSIs) and a test set (37 WSIs). The adaptation set originates from three annotations per subclass. To give an example, patches from the subclass *sarcomatoid* were extracted from three annotations, each in a different WSI. The test set contains 13,963 image patches of class *tumor*, 22,739 image patches of class *healthy*, and 2126 image patches of class *necrosis*.

### Prototypical few-shot classification

**Fig. 4:**
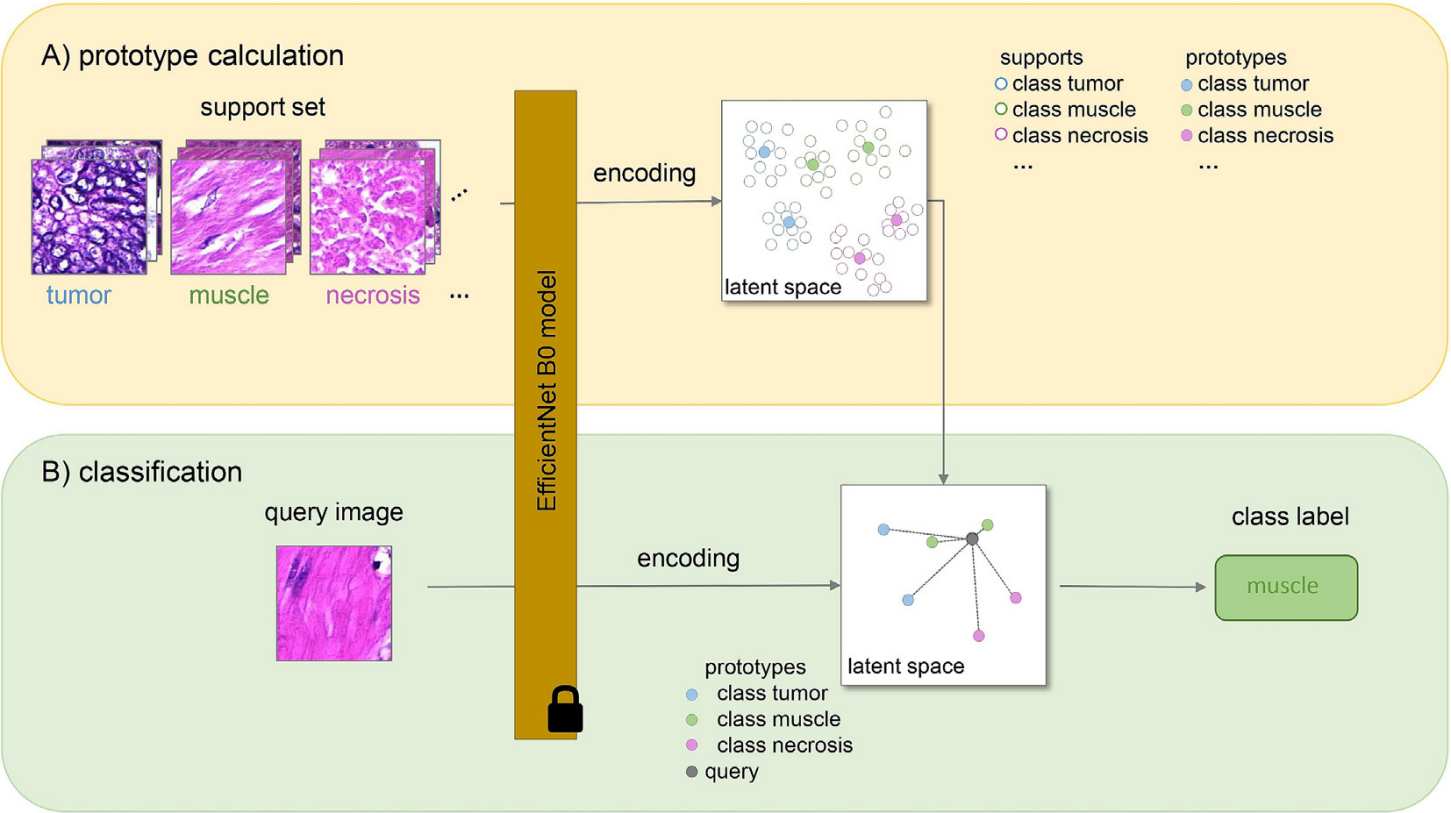
Prototypical few-shot models rely on an explicit class representation in latent space by so-called “prototypes”. For the calculation of the prototypes, some user-provided example images, referred to as “supports”, are required for each class. These supports are propagated through a CNN model, resulting in a feature vector per image. Class prototypes are then computed by applying the k-means algorithm to all supports of one class. At run-time, a query image is encoded by the same CNN model. The class label of the closest prototype in latent space is assigned to the query image.

Prototypical few-shot networks were introduced by Snell et al.^26^ Their key idea is to use a CNN architecture to encode each image patch and afterwards perform the classification in latent space based on the similarity to so-called class prototypes (Fig. 4).

In the encoding process, a fixed CNN model is used to compute a multidimensional feature vector for each image patch. It is also possible to employ a vision transformer instead of a CNN.^7^ In this work, we compared various popular CNN models (Subsection Comparison of different network architectures) and omitted in each case the original fully connected and softmax layers to obtain a feature vector instead of a class label probability. The feature vector represents the input image patch in the latent space. Further details are provided in Subsection Comparison of different network architectures.

The target classes, to which image patches should be assigned, are represented by one or multiple prototypes in the latent space. Prototypes are in turn derived from a set of user-provided example image patches, which are denoted “supports”. In contrast to Snell et al.^26^ who used only one prototype per class—in their case defined as the average of the corresponding support feature vectors—we represented each class by multiple prototypes. Especially in histopathology, where tissue classes such as *tumor* exhibit a high level of heterogeneity, a multiprototype representation was shown to be beneficial.^8^ In this work, we applied a k-means clustering^12^ on the supports of each class to obtain its multiprototype representation.

In order to classify unknown query images, they have to be encoded by the same CNN model that encoded the supports. Afterwards, the similarity of a query feature vector to all prototypes is measured and the class label of the most similar prototype is assigned to the query (Fig. 4). In this work, the squared Euclidean distance was used as similarity measure.

### Training procedure of few-shot models

Given is a training data set $D={\left(x_{i},y_{i}\right)}_{i=1}^{N}$ of N pairs of image patches $x_{i}\in {\mathbb{R}}^{n}$ and corresponding class labels $y_{i}\in \left\{1,\ldots ,C\right\}$. The image patches are encoded with a CNN model $f_{\theta }:{\mathbb{R}}^{n}\to {\mathbb{R}}^{d}$, with parameters θ to generate a representation $R={\left(f_{\theta }\left(x_{i}\right),y_{i}\right)}_{i=1}^{N}$ of the training set in the latent space.

We apply an episodic procedure, denoted as $N^{c}$-way $N^{s}$-shot. In each episode, a subset C of classes is randomly chosen from the whole set of classes: $C\subseteq \left\{1,\ldots ,C\right\}$. Then, for each class c∈C $N^{s}$ supports and $N^{q}$ queries are drawn. The supports are used to calculate prototypes, whereas the queries are referred to for the loss computation. The specific settings for each experiment are provided in Section Results and discussion.

### Data augmentation

Based on earlier experiments with classical CNN models on the multiscanner data set,^19^^,^^20^ four different augmentations were applied randomly during training that reflect the variances introduced by differences in the staining and digitization processes (Fig. 1). One is a domain specific augmentation technique introduced by Tellez et al.,^30^ where the HE components are separated by color deconvolution and afterwards varied independently. Two further augmentations were applied in the HSV color space: in one case, only the hue values were altered and in the other case, only the saturation values were changed. Another augmentation consisted of applying a Gaussian blur filter, simulating out-of-focus regions introduced during scanning.

### Interactive creation of classifiers

**Fig. 5:**
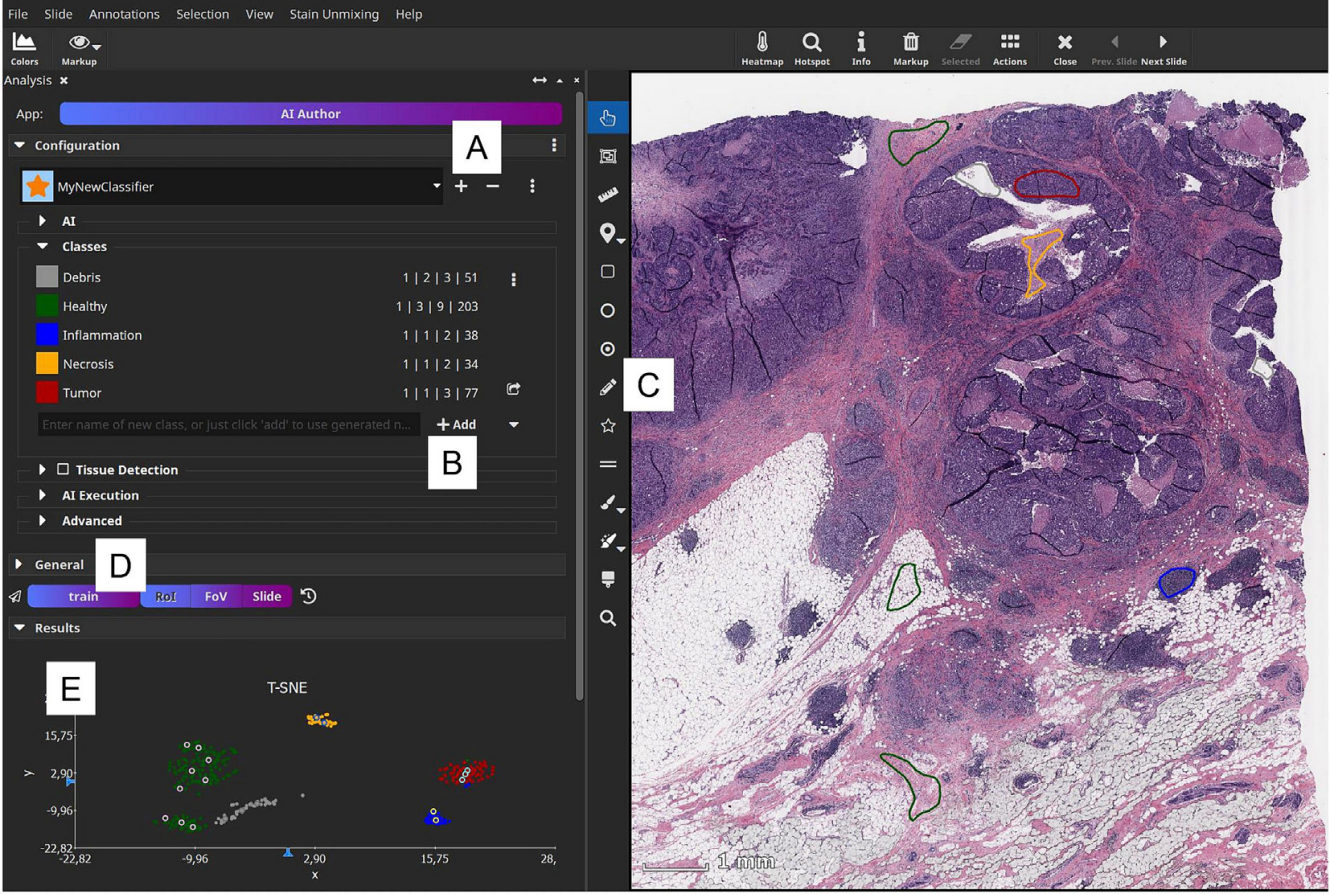
User interface of the MIKAIA software (version 1.4.0) to create an adapted few-shot classifier. First, a new classifier is added (A) and afterwards classes can be named and added (B). Then, example annotations have to be provided for each class (C). Clicking “Train” triggers the computation of the supports' feature vectors with the DNN and calculation of the per-class prototypes (D). A two-dimensional projection of the resulting feature vectors is displayed in (E). The supports of each class are color-coded according to their class labels.

The main advantage of prototypical few-shot models is that they can be adapted to new tasks without retraining of the weights of the underlying network architecture. The DNN is merely used as a feature extractor and remains unchanged when applied to new tasks. Only the class prototypes have to be exchanged (Fig. 4), i.e., the combination of the (constant) feature extraction model and the set of classes and their prototypes represent a new “AI” that was adapted to a new task. Therefore, only a few annotations for each class in a small set of WSIs are required. Based on these annotations, support image patches for each class can be generated. This workflow of annotating some examples, adapting a prototypical few-shot model and applying it to WSIs is realized in the MIKAIA software (Fig. 5). Moreover, the model can be adapted iteratively. Starting with a few annotations and a first version of prototypes, the few-shot model can be applied to a selected region. Subsequently, incorrectly classified regions can be annotated correctly and included in the prototype calculation.

The adaptation of the few-shot model in experiment in Subsection Adaptation to urothelial carcinoma was performed using MIKAIA (version 1.2.0). A simple workflow was applied without iterative adjustment of the few-shot model: all annotations were created first, and afterwards, the prototypes were calculated by using k-means clustering (with *k* = 6), i.e., each class is represented by six prototypes. Especially for the iterative workflow, where a user first creates annotations, then tests the classifier, and creates additional “corrective” annotations in misclassified regions, a prototype calculation is desired that utilizes the annotator's intention behind individual annotations. Instead of pooling all supports from all annotations and applying k-means clustering, one could calculate one prototype for each annotation. A comparison of these two methods can be found in Kletzander et al.^18^

## Experimental settings

This section describes in detail the various experiments we conducted to investigate the presented few-shot approach's robustness and its adaptability to a new task. All few-shot models were trained using the TensorFlow framework (version 2.6).

### Comparison with classical CNN and influence of data augmentation

One goal of this experiment was to compare the performance of a few-shot model with a classical CNN model. A second goal was to investigate the robustness of the models against domain shifts caused by different stain protocols and scanners.

This experiment was performed with EfficientNet B0 few-shot models. The reference was a classical approach that also uses EfficientNet B0 but with fully connected layer and a softmax layer on top. All models were trained on the primary training set, both with data augmentation (as described in Subsection Data augmentation) and without data augmentation. During training, all models were evaluated on the primary validation set after each epoch. From each training run, the model at the epoch with the best validation loss was chosen. Each model was trained three times.

The few-shot models were trained for 20 epochs each consisting of 28,000 episodes in a 3 way 20 shot setting ($N^{c}=3)$, ($N^{s}=20$) with 5 queries per class ($N^{q}=5$). For each class 3, prototypes were calculated with the k-means clustering algorithm (*k* = 3). We employed CORELloss^16^ and the Adam optimizer with slightly different learning rates between 10^-5^ and 1.5×10^-5^ as well as exponential decay. All color augmentations were applied with a probability of 0.5 and the blur augmentation with 0.3. In a post-processing step after the training was finished, the class prototypes for the evaluation were calculated. The support image patches were randomly selected from the primary scanner adaptation set and subsequently used in all the experiments. For each class, 1000 image patches were used and three prototypes calculated with the k-means clustering algorithm (*k* = 3).

The CNN models were also trained for 20 epochs with a batch size of 105. We used a categorical Cross-Entropy loss and Adam optimizer with a learning rate of 10^-5^ and exponential decay. Again, all color augmentations were applied with a probability of 0.5 and the blur augmentation with 0.3.

Finally, all models were evaluated on the six test sets of the multiscanner database (Fig. 3). The results were averaged over all three models of each setting.

### Comparison of different network architectures

The goal of this experiment was to investigate the robustness of few-shot models using different network architectures as a feature extractor on the multiscanner database. Moreover, their inference time was compared.

The following network architectures were chosen for the comparison: EfficientNet B0, EfficientNet B3, EfficientNet B4,^29^ Xception,^5^ DenseNet 121,^15^ MobileNet V2,^25^ ResNet 50,^13^ Inception V3,^28^ and QuickNet.^11^ For better comparability, we wanted to use the same dimension of the latent space for all few-shot models and set it to 512. Therefore, all architectures that originally use a latent space with different dimensions were adapted with a fully connected layer resulting in the required dimension of 512. The training procedure for all models included data augmentation and was the same as in Subsection Comparison with classical CNN and influence of data augmentation and so was the prototype selection and evaluation on the six scanner test sets (Fig. 3). One exception was the learning rate, which varied here between 10^-5^ and 10^-4^. Moreover, due to memory constraints, the EfficientNet B3 few-shot model was only trained in a 3 way 15 shot setting ($N^{c}=3$, $N^{s}=15$) with 5 queries per class ($N^{q}=5$) and the EfficientNet B4 few-shot model only in a 3 way 12 shot setting ($N^{c}=3$, $N^{s}=12$) with 4 queries per class ($N^{q}=4$). Finally, all models were evaluated on the six test sets of the multiscanner database (Fig. 3). The results were averaged over all three models (resulting from the three training runs per architecture) of each architecture.

A comparison of the inference time was performed with a NVIDIA GeForce GTX 1060 GPU with 6 GB memory using the TensorFLow 2.3C API with a batch size of 30 and averaging over 135 batches.

### Investigation of different adaptation sets

This experiment aimed to investigate the influence of class prototypes created from different adaptation sets on the classification performance and robustness of the few-shot model.

To this end, we used the few-shot models with an EfficientNet B0 network architecture and trained with data augmentation from the experiment described in Subsection Comparison with classical CNN and influence of data augmentation. However, we used different class prototypes originating from the six different adaptation sets of the multiscanner data set (Fig. 3). All class prototypes for one few-shot model are derived from only one scanner adaptation set. For each class, 1000 image patches were selected randomly. Two exceptions are the classes inflammation and mucus from scanner B, where due to the scanner's lower resolution, only fewer patches were available. Here, only 678 image patches with class label *inflammation* and 983 image patches with class label *mucus* are available. Three prototypes per class were calculated applying k-means clustering (*k* = 3) to the feature vectors of all image patches of the respective class.

The evaluation of the few-shot models with the different sets of class prototypes was performed on the multiscanner database (Fig. 3). The results were averaged over all three models (resulting from three independent training runs) with prototypes from the same scanner adaptation set.

### Evaluation on multicenter data set

Domain shifts might not only be introduced by the use of different scanners for digitization but also by differences in staining protocols or workflows in the preparation of tissue section between different sites. Therefore, the goal of this experiment was to compare the classification performance on data from different centers.

Again, the EfficientNet B0 architecture was chosen for this experiment using one few-shot model including prototypes and one classical CNN model trained with data augmentation from the first experiment (Subsection Comparison with classical CNN and influence of data augmentation). Both models were evaluated on the multicenter data set that comprises data from two separate hospitals as well as from the inherently multicenter TCGA.

### Adaptation to urothelial carcinoma

Prototypical few-shot models can be easily adapted to new tasks by exchanging the prototypes. Whereas in the previous experiments we regarded the robustness of the few-shot models, here we investigate the adaptation to a different tumor entity. As the feature extraction network was originally trained on HE-stained colon section, we opted to test on an entity outside of the gastrointestinal tract and selected to test on urothelial carcinoma.

The experiment was performed with one of the few-shot models with an EfficientNet B0 architecture that was trained with data augmentation on the primary training set in the experiment described in Subsection Comparison with classical CNN and influence of data augmentation. Class prototypes for the three classes: *tumor*, *healthy*, and *necrotic* were calculated based on all images patches in the urothelial adaptation set, again using k-means clustering, this time with *k* = 6 prototypes per class. Afterwards, this model was evaluated on the urothelial test set.

## Results and discussion

The results of all conducted experiments are provided and discussed in the following subsections.

### Comparison with classical CNN and influence of data augmentation

**Fig. 6:**
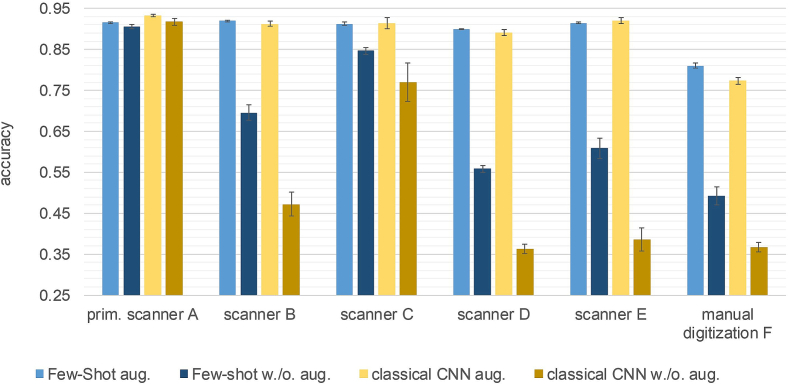
Comparison of the accuracy obtained by few-shot models and classical CNN models trained with and without data augmentation on the multiscanner test set. The training was repeated three times for each setting. The results were averaged over the three resulting models and the error bars correspond to the standard deviation.

Fig. 6 shows the accuracy on all six scanner test sets obtained with the few-shot EfficientNet B0 models and the classical CNN models. The models trained without data augmentation perform well on the primary scanner test set but they do not cope with the domain shift introduced by different scanners. This is indicated by the significantly lower accuracy values on all other scanner test sets (B–F) with an average decrease of 25% points for the few-shot models and 41% points for the CNN models. Applying data augmentation during the training process successfully increases the models' robustness. This is remarkable because all models were trained on the primary dataset only, in which all sections were from the same clinic and digitized with only one scanner. The chosen augmentations effectively mimic the domain shifts introduced by different scanners. These models obtained a high accuracy of around 90% on all foreign automated scanner test sets (B–E), which is comparable to the accuracy achieved on the primary scanner test set. An exception is the test set obtained by manual digitization (F), where a performance drop occurred. However, here they also achieve a significantly higher accuracy than the models trained without data augmentation. Notably, the overall performance of the few-shot model and the classical CNN model trained with data augmentation are very similar.

### Comparison of different network architectures

**Fig. 7:**
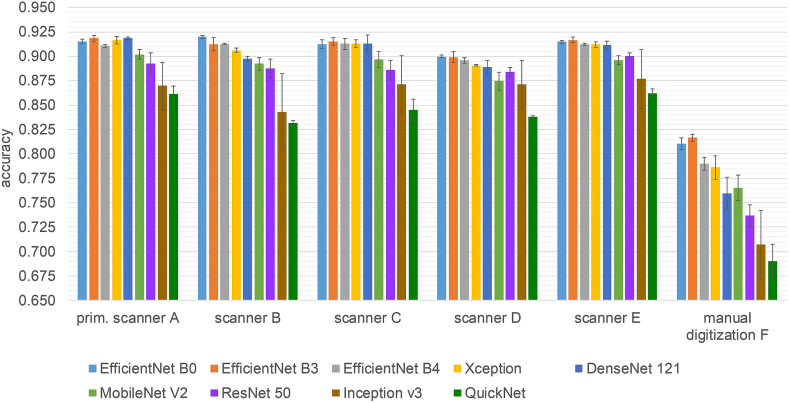
Comparison of the accuracy obtained by few-shot models with different network architectures on the multiscanner test set. The training was repeated three times for each architecture. The results were averaged over the three resulting models and the error bars correspond to the standard deviation.

In Fig. 7, the accuracy values obtained by few-shot models with different network architectures on the multiscanner test set are shown. The overall performance of the different network architectures is very similar. All variants obtain the lowest accuracy on the “manual digitization test set” and the accuracy obtained on the “scanner D test set” is slightly lower (1–2% points) than on the other scanner test sets, both by the few-shot and classical CNN models. For each architecture, three models were trained and the results averaged. Especially all EfficientNet architectures obtained stable results indicated by values of 0.007 and lower for the standard deviation. The highest variance between different training runs shows the Inception V3 architecture. Also the standard deviation of all architectures are higher on the “manual digitization test set”.

**Fig. 8:**
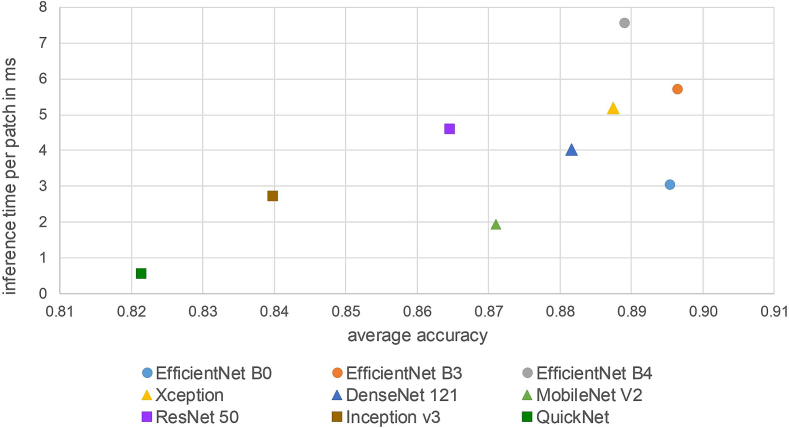
Inference time per image patch in ms against averaged accuracy over all six scanner test sets for few-shot models with different network architectures.

Another important aspect in the assessment of the different few-shot models is their inference time. WSIs typically have a huge size resulting in several tens of thousands image patches per WSI. Fig. 8 shows the inference time for each network architecture against their accuracy averaged over all six scanner test sets. The three EfficientNet variants obtained the highest overall accuracy. We speculate that the unexpected lower overall accuracy of the few-shot model using EfficientNet B4 compared to the other two EfficientNet variants may be caused by our adaptation of this architecture to reduce the dimension of the resulting feature vector to 512 or by the lower number of supports used during the episodic training. The best trade-off between accuracy and inference time is achieved by EfficientNet B0. Only MobileNet V2, Inception V3, and QuickNet are faster, but their overall accuracy is more than 2% points lower.

### Impact of different adaptation sets

**Fig. 9:**
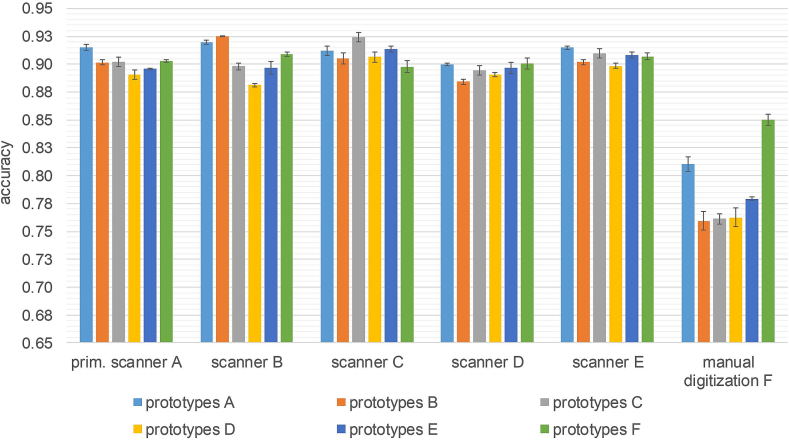
Comparison of the accuracy obtained by few-shot EfficientNet B0 models on the multiscanner test set with different class prototypes each derived from one scanner adaptation set. The results were averaged over all three EfficientNet B0 models (resulting from three independent training runs) with prototypes from the same scanner adaptation set. The error bars correspond to the standard deviation.

Fig. 9 shows the results obtained with prototypes calculated from the different scanner adaptation sets (Fig. 3) for few-shot models with an EfficientNet B0 architecture. Overall, the models that use prototypes which were calculated on the target scanner, i.e., where adaptation and test images stem from the same scanner, tend to achieve the best accuracy. Exceptions are scanners D and E, but results for these two scanners are close together with a difference of 1.6% points between the lowest and highest accuracy. The use of scanner-specific prototypes leads to the highest improvement in accuracy for the “manual digitization test set” underlining again that the WSIs generated by manual digitization possess some characteristics that differ strongly from the other scanner data. All in all, the few-shot models proved their robustness when using prototypes derived from scanner data that they were not trained with an average absolute deviation in accuracy compared to the primary prototypes of 1.8% points.

### Evaluation on colon adenocarcinoma multicenter data set

Table 2 and Table 3 show the evaluation results of a few-shot model with the EfficientNet B0 architecture and the corresponding classical approach on the multicenter data set and the corresponding results on the primary test set from Center 1. The achieved overall accuracy is similar on all data sets and for both models. Also, the precision, recall, and F1 values averaged over the seven tissue classes with equal weight achieved by the respective models on the multicenter data sets are close to or even higher than the respective values on the primary set except for the average precision and F1 value of the classical CNN model on the TCGA test set (Center 4). The difference was mainly caused by a worse precision of the most underrepresented class in the TCGA test set. The model achieved only a precision of 0.61 for the class *necrosis* compared to 0.91 precision for the same class in the primary test set that stems only from Center 1. Hereby, the majority (82%) of incorrect predictions for necrosis resulted from image patches of only four WSIs.

**Table 2:** Evaluation results obtained by a few-shot model using EfficientNet B0 and a classical EfficientNet B0 model on the colon adenocarcinoma multicenter test set. The precision, recall, and F1 values are averaged over all seven tissue classes, where each class was weighted equally, independent of the number of image patches within the class. The standard deviation is denoted next to the average value for each metric and data set. Both models were trained only with data from the primary scanner.

	Few-shot		

	Avg. precision	Avg. recall	Avg. F1

Primary set center 1	0.80 ± 0.17	0.93 ± 0.02	0.85 ± 0.11
Center 2	0.90 ± 0.10	0.92 ± 0.09	0.90 ± 0.06
Center 3	0.87 ± 0.11	0.93 ± 0.04	0.90 ± 0.06
Center 4	0.79 ± 0.18	0.91 ± 0.07	0.83 ± 0.11

	Classical CNN		

	Avg. precision	Avg. recall	Avg. F1

Primary set center 1	0.89 ± 0.09	0.90 ± 0.06	0.89 ± 0.06
Center 2	0.91 ± 0.08	0.91 ± 0.07	0.91 ± 0.05
Center 3	0.91 ± 0.07	0.88 ± 0.11	0.89 ± 0.08
Center 4	0.82 ± 0.17	0.87 ± 0.10	0.83 ± 0.12

**Table 3:** Overall accuracy obtained by a few-shot model using EfficientNet B0 and a classical EfficientNet B0 model on the colon adenocarcinoma multicenter test set. Both models were trained only with data from the primary scanner. The 95% confidence interval is denoted for each data set and both models.

	Overall accuracy	
	Few-shot	Classical CNN
Primary set center 1	0.922 ± 0.010	0.930 ± 0.013
Center 2	0.937 ± 0.035	0.946 ± 0.030
Center 3	0.933 ± 0.020	0.950 ± 0.015
Center 4	0.911 ± 0.030	0.910 ± 0.030

These overall metrics indicate that both models are robust against the domain shift introduced by data from different centers. However, taking a closer look at the class *tumor* reveals another challenge. Table 4 provides the class-specific precision, recall, and F1-Score for the class *tumor*. While the precision values are very similar to the ones achieved on the primary test set, especially with regards to Center 2 and Center 4, the recall shows a significant drop for these two centers. Also, the width of the 95% confidence interval of the recall is noticeably higher for Center 2. This is partly due to the fact that there are only 16 WSIs in total in this data set and only 13 of these contain tumorous tissue. In particular, WSIs with tumor subtypes (e.g., mucinous adenocarcinoma with signet-ring cell components) that were underrepresented in the training set and not present in the adaptation set of the few-shot model showed a low tumor recall. This demonstrates the importance of a representative training or prototype set where all subtypes that should be recognized by the model are present.

**Table 4:** Evaluation results for the class *tumor*, obtained by a few-shot model using EfficientNet B0 and a classical EfficientNet B0 model on the colon adenocarcinoma multicenter test set. Both models were trained only with data from the primary scanner. The 95% confidence interval is denoted for each metric and data set.

	Few-shot (class *tumor*)		

	Precision	Recall	F1

Primary set center 1	0.98 ± 0.03	0.94 ± 0.02	0.96 ± 0.01
Center 2	0.99 ± 0.04	0.73 ± 0.17	0.84 ± 0.12
Center 3	0.91 ± 0.04	0.91 ± 0.04	0.91 ± 0.03
Center 4	0.96 ± 0.02	0.75 ± 0.06	0.85 ± 0.04

	Classical CNN (class *tumor*)		

	Precision	Recall	F1

Primary set center 1	0.97 ± 0.02	0.95 ± 0.02	0.96 ± 0.01
Center 2	0.97 ± 0.04	0.85 ± 0.11	0.91 ± 0.08
Center 3	0.93 ± 0.03	0.87 ± 0.05	0.90 ± 0.04
Center 4	0.97 ± 0.01	0.70 ± 0.07	0.82 ± 0.05

### Adaptation to urothelial carcinoma

The results of the adapted few-shot model applied to the urothelial test set are given in Table 5. Especially for the classes *tumor* and *healthy*, high recall and precision values of 90% and above are obtained. Although the urothelial test set covers a broad range of different tumor subtypes, the recall of the class *tumor* is 90.0%. While the adaptation set also contained annotations from all subtypes, this shows that a class represented by multiple prototypes can accurately describe a heterogeneous tissue type. The overall accuracy on the urothelial test set is 93.6%.

**Table 5:** Evaluation result on urothelial test set achieved with adapted few-shot model. The 95% confidence interval is denoted for each metric and tissue type.

Class	Precision	Recall	F1
Tumor	0.932 ± 0.025	0.900 ± 0.033	0.916 ± 0.022
Healthy	0.950 ± 0.047	0.958 ± 0.029	0.954 ± 0.026
Necrosis	0.811 ± 0.109	0.931 ± 0.067	0.867 ± 0.065

These results demonstrate that the few-shot model where the feature extraction backbone was trained on colon adenocarcinoma from a single center (Center 1) could be adapted successfully to the analysis of a heterogeneous urothelial carcinoma data set, comprising six tumor subtypes, by providing only a few annotations (refer Subsection Urothelial cancer data set for details).

## Conclusion and future work

Prototypical few-shot models successfully counter the label scarcity challenge in histopathology. They can be adapted to new tasks by providing only a small set of annotations and do not require a retraining of any network weights. This avoids the need for choosing fine-tuning hyperparameters as well as the sophisticated task of preventing overfitting given only a small set of labeled data. Moreover, the determination of new prototypes can be performed rapidly, as it only requires inference of a small support set and the application of a clustering algorithm. In conjunction, this renders prototypical few-shot models an ideal technology for realizing an interactive “AI authoring” workflow that can be driven by non-technical users, as no human inputs, except for a small amount of example annotations, are required and new models can be adapted very quickly. The realization of an interactive user interface is illustrated in Fig. 5.

We demonstrated that a set of domain specific data augmentations during training ensures robustness of the prototypical few-shot models against domain shift introduced by differences in the digitization and staining process, although all training data originated from one scanner and one center. Hence, we achieved an accuracy of about 90% on the primary data set (which the few-shot feature extractor was trained on), as well as on all automated scanner sets and an even higher accuracy on the colon adenocarcinoma multicenter data set. This solution, in contrast to other domain generalizations such as stain normalization or style transfer GANs, has the advantage that it does not pose an additional computational burden at run-time (inference time) and therefore favors higher throughput.

However, the biological diversity, e.g., different cancer subtypes, of the given tasks also has to be taken into account. The evaluation on the multicenter data set revealed a decreased recall for the class *tumor* on WSIs in the test set containing subtypes that were absent or underrepresented in the support set. Experiments with a more fully representative support set will be a next step in our future research. In situations where this is not feasible, an alternative solution would be to reliably detect out-of-distribution data, i.e., data that were not present in the training phase.

The adaptation of our prototypical few-shot model to a new task, namely the classification of tissue sections containing urothelial cancer, achieved very promising results with an overall accuracy of 93.6% and a F1-score of 91.6% for the class *tumor* despite the fact that the encoder was only trained on a single task (albeit with a loss function, COREL, that rewards wide-spread utilization of the latent space, which in turn benefits generalization). Future work will thus incorporate training the feature extractor on a multitask data set. When a new task involves a different stain, such as IHC instead of HE, or is better solved at a significantly higher or lower magnification, the features produced currently will likely prove to be suboptimal. We have yet to investigate how strong this effect is and whether the current feature extraction network can be trained to generalize also in these dimensions or whether it would be beneficial to provide multiple feature extractors and have the user choose which one best fits their new task.

Moreover, our implemented workflow for creating new few-shot classifiers also enables an iterative procedure of providing annotations, adapting class prototypes and afterwards applying the classifier to WSIs. This can also be regarded as an *active learning* system where the user provides additional “corrective” annotations in areas that were misclassified with the classifier of the previous iteration. Within this experiment, only a simple workflow where first all annotations were provided was investigated. Therefore, future experiments will incorporate the iterative procedure.

Lastly, the models compared in this work were all patch classification models. To get around the typical checkerboard-like result, we optionally run a superpixel segmentation first, then classify some or all patches contained in a superpixel and assign the majority label to the entire superpixel.^35^ However, patch classification models are of limited use when a fine-grained segmentation is required. We aim to extend this few-shot methodology from (coarse) patch classification to pixel-accurate segmentation and have already published first results,^10^ but plan to carry out further research.

## CRediT authorship contribution statement

**Petr Kuritcyn:** Conceptualization, Methodology, Soft- ware, Investigation, Writing - Original Draft. **Rosalie Kletzander:** Methodology, Software, Investigation, Writing - Review & Editing. **Sophia Eisenberg:** Data Curation. **Thomas Wittenberg:** Funding aquisition, Writing - Review & Editing. **Volker Bruns:** Conceptualization, Writing - Original Draft. **Katja Evert:** Resources, Data Curation, Writing - Review & Editing. **Felix Keil:** Resources, Data Curation, Writing - Review & Editing. **Paul K. Ziegler:** Resources, Data Curation, Writing - Review & Editing. **Katrin Bankov:** Resources, Data Curation, Writing-Review & Editing. **Peter Wild:** Writing - Review & Editing. **Markus Eckstein:** Resources, Data Curation, Writing - Review & Editing. **Arndt Hartmann:** Writing - Review & Editing. **Carol I Geppert:** Resources, Data Curation, Writing - Review & Editing. **Michaela Benz:** Conceptualization, Supervision, Writing - Orinigal Draft.

## Declaration of competing interest

The authors declare the following financial interests/personal relationships which may be considered as potential competing interests:

The MICAIA software developed by Fraunhofer IIS and presented in this manuscript is now a commercially available software. (Peter Kuritcyn, Rosalie Kletzander, Sophia Eisenberg, Thomas Wittenberg, Volker Bruns, Michaela Benz) If there are other authors, they declare that they have no known competing financial interests or personal relationships that could have appeared to influence the work reported in this article.
